# Central retinal vein occlusion post ChAdOx1 nCoV-19 vaccination – can it be explained by the two-hit hypothesis?

**DOI:** 10.1186/s12348-022-00311-4

**Published:** 2022-10-26

**Authors:** Shweta Parakh, Shrey Maheshwari, Shrutanjoy Das, Hans Vaish, Gaurav Luthra, Rupesh Agrawal, Vishali Gupta, Saurabh Luthra

**Affiliations:** 1 Drishti Eye Institute, Dehradun, India; 2 Meher Hospital, Dehradun, India; 3 National Healthcare Group Eye Institute, Tan Tock Seng Hospital, Singapore, Singapore; 4 Lee Kong Chian School of Medicine, Nanyang Technological University, Singapore, Singapore; 5 Advanced Eye Centre, Postgraduate Institute of Medical Education and Research, Chandigarh, India; 6 Present Address: Drishti Eye Institute, 16, Subhash Road, Astley Hall, Dehradun, Uttarakhand 248001 India

**Keywords:** ChAdOx1 nCoV-19 vaccine, Central retinal vein occlusion (CRVO), Vaccine-induced immune thrombotic thrombocytopenia (VITT), Anti-PF4 antibodies, SARS-CoV-2, Two-hit hypothesis

## Abstract

**Purpose:**

To report a case of central retinal vein occlusion (CRVO) seven days following the first dose of ChAdOx1 nCoV-19 vaccine and propose a hypothesis for the possible underlying pathogenesis.

**Observation:**

A 31-year-old male presented with CRVO with cystoid macular edema, one week after receiving his first ChAdOx1 nCoV-19 vaccine dose. Apart from mild hyperhomocysteinemia, no major thrombophilic or systemic risk factors were found. Anti-platelet factor 4 antibodies, specific for vaccine-induced immune thrombotic thrombocytopenia, were also negative. However, he tested strongly positive (> 250 U/mL) for severe acute respiratory syndrome coronavirus 2 (SARS-CoV-2) IgG spike antibodies, 2 weeks post the first dose – suggestive of a prior subclinical infection.

**Conclusion:**

COVID-19 is known to be associated with an altered host one-carbon metabolism resulting in hyperhomocysteinemia. We hypothesize that a prior subclinical infection with COVID-19, the first hit, may have led to hyperhomocysteinemia in our patient and vaccination must have been the second hit that triggered the thrombotic event. Further studies, including correlation of thrombotic complications with IgG antibody titres post-vaccination, are essential in order to better understand the pathogenesis of such events.

## Introduction

Vaccines against severe acute respiratory syndrome coronavirus 2 (SARS-CoV-2) are the mainstay to fight the coronavirus disease 2019 (COVID-19) pandemic. Eight vaccines have been approved for emergency or full use by at least one regulatory authority recognized by the World Health Organization (WHO) as follows: Pfizer–BioNTech (BNT162b2/COMIRNATY), Oxford–AstraZeneca (AZD1222 Vaxzevria / ChAdOx1-nCoV-19), Sinopharm BIBP (SARS-CoV-2 Vaccine [Vero Cell], Inactivated [lnCoV]), Moderna (mRNA 1273), Janssen (Ad26.COV2.S), CoronaVac, Covaxin and Novavax (NVX-CoV2373/Nuvaxovid). Five others are under assessment by the WHO: Sputnik V, Sinopharm WIBP, Convidecia, Sanofi–GSK and SCB-2019 [1].

Though there are case reports and series [2–7] of vascular occlusions occurring post vaccination against SARS-CoV-2, very few patients had a history of prior clinical COVID-19 infection. We report a case of central retinal vein occlusion (CRVO) in a young male, occurring one week post the first dose of ChAdOx1 nCoV-19 vaccination. He was found to have mild hyperhomocysteinemia and markedly elevated IgG antibody titres against SARS-CoV-2 spike protein, suggestive of a prior sub-clinical infection. It is now known that SARS-CoV-2 significantly stresses the host’s one-carbon metabolism and simultaneously increases demand but reduces supply of methyl-groups [8]. This biochemical imbalance leads to elevated homocysteine levels, that have been implicated in increased lipid peroxidation, endothelial damage and thromboembolism [9, 10]. The two-hit hypothesis seems to be a reasonable model to explain the probable course of events in our case. It explains how successive inflammatory or ischemic insults can synergistically lead to an inappropriate immune response, often resulting in organ damage or dysfunction [11]. We hypothesize that a possible prior subclinical infection with COVID-19 in our patient may have led to hyperhomocysteinemia—the first hit—and vaccination must have been the second hit that finally precipitated the thrombotic event.

## Case

**Fig. 1:**
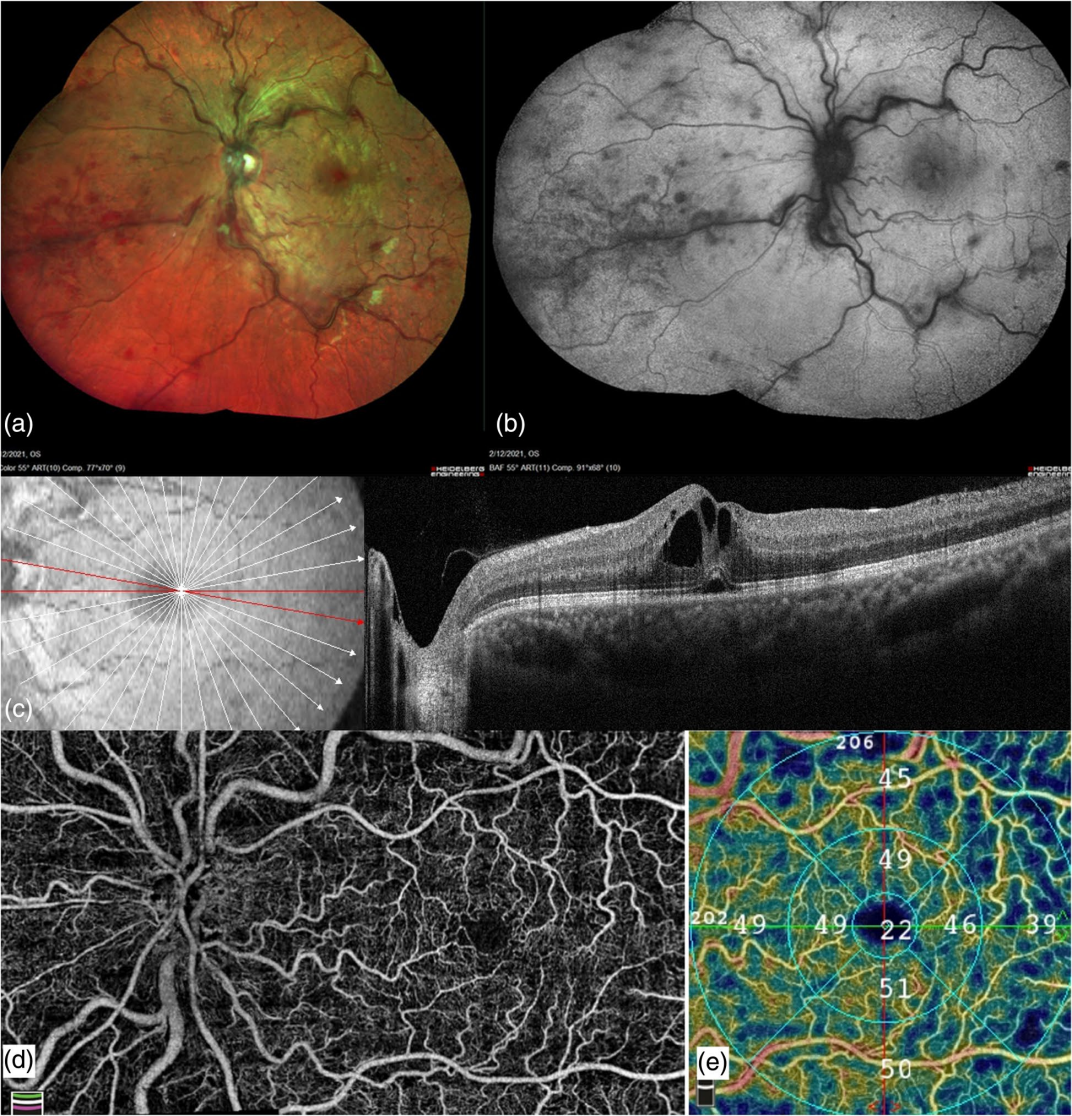
Multimodal imaging of left eye central retinal vein occlusion post ChAdOx1 nCoV-19 vaccination. At the first visit (one week post vaccination). **a**, **b** Multicolor fundus imaging and corresponding short-wave fundus autofluorescence showing disc hyperemia, macular edema, multiple retinal hemorrhages and dilated tortuous veins. **c** Structural spectral domain optical coherence tomography (SD-OCT) showing cystoid macular edema and neurosensory detachment. **d** Montage optical coherence tomography angiography (OCTA) image of the optic disc and macula showing flow void areas. **e** Reduction of vessel density in superficial capillary plexus on OCTA

A 31-year-old male presented in February 2021 with complaints of sudden onset, painless diminution of vision in the left eye (OS) for 2 days. Best corrected visual acuity (BCVA) was 6/6 in the right eye (OD) and 6/9 OS. Intraocular pressure was normal in both eyes (OU). Pupils were equal, round and reactive to light OU. Anterior segment examination was unremarkable OU. Dilated fundus examination was within normal limits OD. Fundus and corresponding short-wave fundus autofluorescence (SW-FAF) (Heidelberg Retina Angiograph, Heidelberg Engineering, Heidelberg, Germany) showed markedly dilated tortuous veins, superficial and deep intraretinal hemorrhages in all four quadrants with macular edema, disc hyperemia and few cotton-wool spots OS, suggestive of central retinal vein occlusion (CRVO) (Fig. 1a, b). Spectral-domain optical coherence tomography (SD-OCT) (RTVue XR Avanti, Optovue Inc., Fremont, CA, USA) showed cystoid macular edema (CME) and neurosensory detachment (NSD) with a central macular thickness of 485 microns OS (Fig. 1 c). Flow void areas with reduction in vessel density in the superficial capillary plexus was seen in OS on optical coherence tomography angiography (OCTA) (RTVue XR Avanti, Optovue Inc., Fremont, CA, USA) (Fig. 1d, e).

A week before presenting with CRVO, the patient had received his first dose of ChAdOx1 nCoV-19 AstraZeneca vaccine against Coronavirus disease 2019 (COVID-19). He did not have any known medical history of systemic illness including hypertension, diabetes, dyslipidemia, hypercoagulability disorder or autoimmune disease. Blood pressure was normal. Hemogram including platelet count, erythrocyte sedimentation rate and C-reactive protein were normal. HbA1c and lipid profile were within normal limits. Serum homocysteine was mildly elevated at 22.19 micromol/L. A hypercoagulability screening panel, including prothrombin time, activated partial thromboplastin time, serum fibrinogen, D-dimer and anti-phospholipid antibodies (anticardiolipin antibodies, lupus anticoagulant antibodies and anti-beta2-glycoprotein-I antibodies – IgM and IgG), Protein C, Protein S levels were all within normal limits. Real-time reverse transcription–polymerase chain reaction (RT-PCR) for SARS-CoV-2 was negative. Anti-platelet factor 4 (anti-PF4) IgG antibody—specific for vaccine induced thrombotic thrombocytopenia (VITT)—was within normal limits (0.14 U/mL) (biological reference interval < 1.00 U/mL). SARS-CoV-2 IgG spike antibody test (SARS-CoV-2 antibodies, nucleocapsid; LabCorp) was strongly positive (> 250 U/mL, biological reference interval < 0.8 negative, > 0.8 positive), 15 days post the first vaccine dose.

**Fig. 2:**
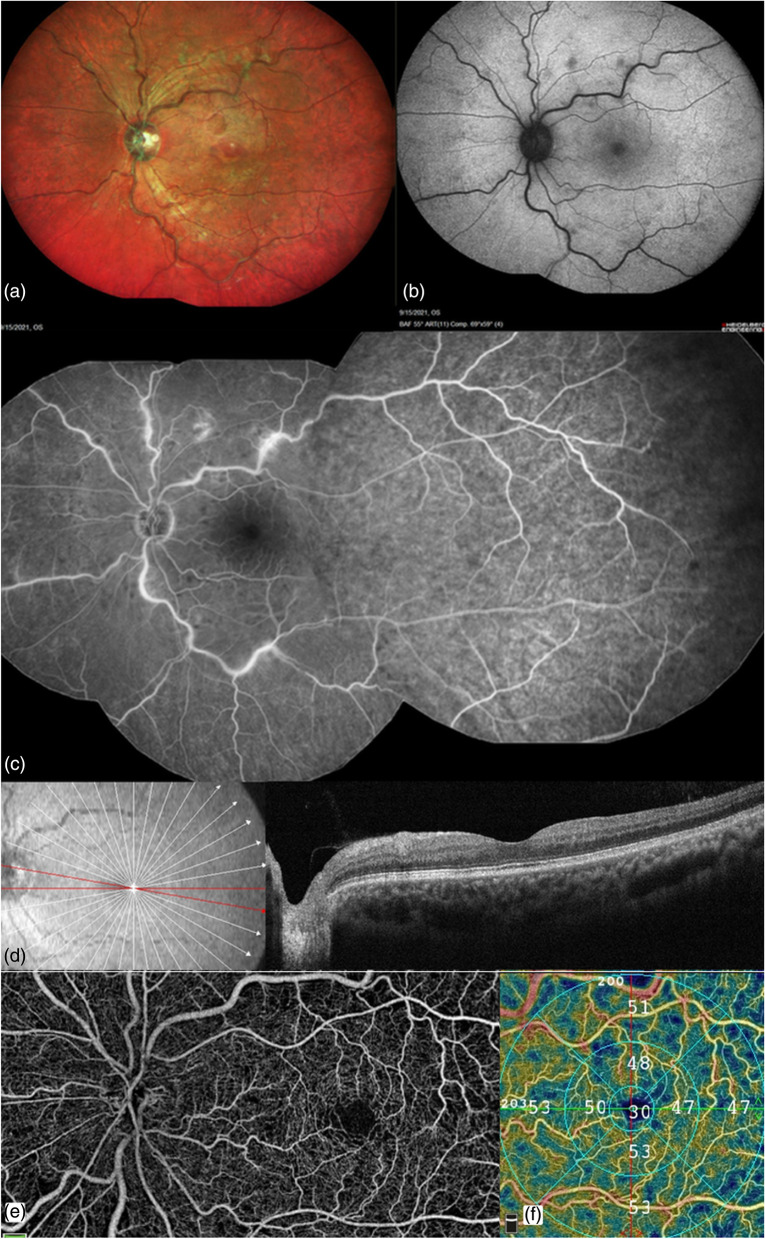
At the 8-month follow-up visit. (Post treatment with four intravitreal anti-VEGF ranibizumab injections). **a**, **b** Multicolor fundus imaging and corresponding short-wave fundus autofluorescence showing reduced intraretinal hemorrhages, cotton-wool spots and venous tortuosity. **c** Fluorescein angiography showing late perivascular staining and capillary non-perfusion in the temporal periphery. **d** Structural spectral domain optical coherence tomography showing resolution of cystoid macular edema and neurosensory detachment. **e** Montage optical coherence tomography angiography (OCTA) of the optic disc and macula showing reduction in flow void areas. **f** Recovery of vessel density in superficial capillary plexus on OCTA

Systemic therapy was initiated with oral folic acid, B_6_ and B_12_ vitamin supplementation by the internist. He was administered four intravitreal injections of anti‑vascular endothelial growth factor (anti-VEGF) ranibizumab (Lucentis®; Genentech, South San Francisco, CA/Roche, Basel, Switzerland) over the next eight months. At the 8-month follow-up visit, BCVA had improved to 6/6 OS. Intraocular pressure was normal OU. Fundus showed reduced intraretinal hemorrhages and cotton-wool spots OS (Fig. 2a, b). Fluorescein angiography (Heidelberg Retina Angiograph, Heidelberg Engineering, Heidelberg, Germany) showed perivascular staining and significant capillary non-perfusion in the temporal periphery OS (Fig. 2c). SD-OCT showed resolution of CME and NSD OS (Fig. 2d). Flow void areas had decreased on OCTA (Fig. 2e,f). Subsequently, he also received three intravitreal anti-VEGF aflibercept (EYLEA; Regeneron Pharmaceuticals, Inc., Tarrytown, New York, USA and Bayer Healthcare Pharmaceuticals, Berlin, Germany) for recurrent CME OS. At the latest follow-up at 18 months, BCVA was 6/9 OS and CME had resolved. All systemic parameters, including serum homocysteine, had normalized and there was no evidence of other systemic thrombotic complications at the end of an eighteen-month follow-up.

## Discussion

The pathogenetic mechanism of central retinal vein occlusion (CRVO) is based on Virchow’s triad of hypercoagulability, endothelial damage and hemostasis—similar to other systemic thrombotic conditions [12]. Elevated prothrombotic markers are a significant risk factor for CRVO in the younger age group. Our patient had no significant predisposing factors except for mildly elevated serum homocysteine (reference intervals—mild 12 to 30 micromol/L, intermediate 31 to 100 micromol/L and severe > 100 micromol/L) [13, 14]. In addition, he was found to have markedly elevated IgG antibody titres against SARS-CoV-2 spike protein as early as 15 days post the first dose. We use the two-hit hypothesis to explain the probable course of events that may have led to the vascular occlusion. The two-hit model suggests that an initial inflammation-triggering event can serve as a priming condition for a limited systemic inflammatory response syndrome. Additional hits are capable of causing an exaggerated immune response [15].

Our patient tested strongly positive (> 250 U/mL) for SARS-CoV-2 IgG spike antibodies, 15 days post the first dose of ChAdOx1 nCoV-19 vaccine. These markedly elevated titres, when temporally correlated with the first dose of the vaccine, suggest that he had possibly been previously infected with COVID-19 prior to vaccination, although he did not show any symptoms. In a recent UK-based study that measured antibody responses in 45,965 adults, who received either the ChAdOx1 or the BNT162b2 SARS-CoV-2 vaccines, it was found that in participants with evidence of prior COVID-19 infection, vaccination increased antibody levels in all age groups [16]. A quantitative analysis of the antibody titres post vaccination revealed the following IgG levels (in ng/ml equivalents, with 95% confidence intervals in parentheses) 28 days post- ChAdOx1 vaccine: 73 (65–81) for 80-year-olds; 94 (87–100) for 60-year-olds; 113 (99–129) for 40-year-olds; and 127 (94–171) for 20-year-olds [16]. These findings are consistent with several previous studies [17–19]. More specifically, the results of a much smaller study on 552 participants in an Indian cohort corroborate with the above studies. It was found that a past history of SARS-CoV-2 infection elicited significantly higher antibody titre compared to SARS-CoV-2 naïve subjects, both after the first (Geometric Mean Titre, GMT, 252.0 vs. 40.8 AU/mL; *p* < 0.001) and second dose (GMT, 302.7 vs. 95.6 AU/mL; *p* < 0.001), irrespective of the type of vaccine received – either ChAdOx1-nCOV (Covishield) and BBV-152 (Covaxin) [20].

In order to explain hyperhomocysteinemia post COVID-19 infection, we refer to McCaddon and Regland’s article which suggests that SARS-CoV-2 significantly stresses the host’s one-carbon metabolism. It simultaneously increases demand but reduces supply of methyl-groups. They proposed that the resultant biochemical alterations – including serine depletion, elevated homocysteine and glutathione depletion—might explain the varied symptoms of COVID-19 that can persist for weeks or months after symptom onset [8]. This condition is called acute post–COVID-19 (from week 5 to week 12), long COVID-19 (from week 12 to week 24), or persistent post–COVID-19 symptoms (lasting > 24 weeks) [21]. The B12-dependent methionine synthase (MS) reaction is highly pertinent to homocysteine metabolism [8]. Vitamin B12 is a cofactor of 2 enzymes present in mammalian cells: methionine synthase and methylmalonyl- CoA mutase enzyme. Deficiency of vitamin B12 results in an increase in methylmalonic acid and homocysteine concentration due to inhibition of methylmalonyl- CoA mutase and methionine synthase, respectively [22]. Kaur et al., [23] proposed that the entry of SARS-CoV-2 facilitated by non-structural protein-14 in the host cell allows the use of cell S-adenosylmethionine for viral RNA capping, resulting in increased homocysteine production and angiotensin-converting enzyme-2 activation, which further augments viral entry into cells [24, 25]. An increased tendency for hypercoagulability and thromboembolism is known to be associated with elevated serum homocysteine [26]. Vitamin B12 therapy is known to reduce oxidative damage and improve microvascular disease associated with hyperhomocysteinemia [27]. Vitamin B12 is an endogenous negative regulator of nuclear transcription factor-B (NF-*κ*B, nuclear factor kappa-light-chain-enhancer of activated B cells) through the regulation of nitric oxide, which plays a key role in regulating the immune response to infection [28, 29]. More detailed analysis of direct and functional markers of Vitamin B12 (e.g., total B12, holotranscobalamin, total homocysteine and methylmalonic acid, total folic acid) are needed to guide the appropriate form of supplementation, with and without COVID-19 infection [30].

In order to explain hyperhomocysteinemia post COVID-19 infection, we refer to McCaddon and Regland’s article which suggests that SARS-CoV-2 significantly stresses the host’s one-carbon metabolism. It simultaneously increases demand but reduces supply of methyl-groups. They proposed that the resultant biochemical alterations – including serine depletion, elevated homocysteine and glutathione depletion—might explain the varied symptoms of COVID-19 that can persist for weeks or months after symptom onset [8]. This condition is called acute post–COVID-19 (from week 5 to week 12), long COVID-19 (from week 12 to week 24), or persistent post–COVID-19 symptoms (lasting > 24 weeks) [21]. The B12-dependent methionine synthase (MS) reaction is highly pertinent to homocysteine metabolism [8]. Vitamin B12 is a cofactor of 2 enzymes present in mammalian cells: methionine synthase and methylmalonyl- CoA mutase enzyme. Deficiency of vitamin B12 results in an increase in methylmalonic acid and homocysteine concentration due to inhibition of methylmalonyl- CoA mutase and methionine synthase, respectively [22]. Kaur et al., [23] proposed that the entry of SARS-CoV-2 facilitated by non-structural protein-14 in the host cell allows the use of cell S-adenosylmethionine for viral RNA capping, resulting in increased homocysteine production and angiotensin-converting enzyme-2 activation, which further augments viral entry into cells [24, 25]. An increased tendency for hypercoagulability and thromboembolism is known to be associated with elevated serum homocysteine [26]. Vitamin B12 therapy is known to reduce oxidative damage and improve microvascular disease associated with hyperhomocysteinemia [27]. Vitamin B12 is an endogenous negative regulator of nuclear transcription factor-B (NF-*κ*B, nuclear factor kappa-light-chain-enhancer of activated B cells) through the regulation of nitric oxide, which plays a key role in regulating the immune response to infection [28, 29]. More detailed analysis of direct and functional markers of Vitamin B12 (e.g., total B12, holotranscobalamin, total homocysteine and methylmalonic acid, total folic acid) are needed to guide the appropriate form of supplementation, with and without COVID-19 infection [30].

ChAdOx1 nCoV-19 vaccines include non-replicating adenoviral vectors, that could induce potent immunological responses due to the presence of viral proteins and stimulation of innate immunity sensors such as toll-like receptors (TLRs) [31]. Endothelial cells express TLRs, which can precipitate intracellular inflammatory responses via mediators such as NF-κB that can eventually modulate vascular permeability and coagulation [32]. Vaccine adjuvants such as alum, emulsions and TLR agonists, produce characteristic profiles with regard to the type (cellular and/or humoral), duration and strength of immune responses [33]. Adjuvants, meant to enhance vaccine-mediated immunity, can lead to autoimmune/inflammatory syndrome in a few susceptible or genetically predisposed individuals [34]. The response of vascular endothelial cells and their constant contact with immune mediators are vital in understanding the process through which successive insults in a two-hit model can cause tissue injury [35].

In a recent multinational case series of 40 centres, seventy patients were diagnosed with an ocular inflammatory event within 14 days of COVID-19. Most common events reported included anterior uveitis (*n* = 41, 58.6%), followed by posterior uveitis (*n* = 9, 12.9%) and scleritis (*n* = 7, 10.0%). These were mild non-sight threatening events that were based on a temporal association that did not prove causality [36]. Commonly proposed mechanisms to explain post-vaccine inflammation include molecular mimicry secondary to similarity between uveal peptides and vaccine peptide fragments apart from antigen-specific cell and antibody-mediated hypersensitivity reactions. In addition, adjuvants included in the vaccines may stimulate innate immunity through endosolic or cytoplasmic nucleic acid receptors [37–41].

Bearing in mind the catastrophic consequences of vaccine-induced immune thrombocytopenia and thrombosis (VITT), our patient was investigated and tested negative for anti-platelet factor 4 (PF4) antibodies. VITT, initially known as thrombosis with thrombocytopenia syndrome (TTS), was first reported by Greinacher et al., post ChAdOx1 nCov-19 vaccination [42]. This oft-fatal condition presents as extensive thrombosis in atypical sites—most commonly in the cerebral venous sinuses—apart from splanchnic, portal and hepatic veins [43]. It has only recently been proven that anti-PF4 antibodies induced by vaccination do not cross-react with the SARS-CoV-2 spike protein. This illustrates that the expected vaccine-induced immune response against the viral spike protein is not the instigating factor for VITT [44].

We report a case of CRVO occurring in close association with and probably precipitated by ChAdOx1 nCoV-19 vaccination. We hypothesize that a possible prior subclinical infection with COVID-19—the first hit – may have led to hyperhomocysteinemia in our patient and vaccination must have been the second hit that triggered the thrombotic event. Although CRVO is not a life-threatening thrombotic phenomenon, it is important for ophthalmologists to be aware of a possible causal and temporal association with COVID-19 vaccination, particularly in young patients with no known systemic risk factors or comorbidities. It is difficult to prove with certainty a direct cause-effect relationship between the vaccination and thrombotic event, as has also been noted in multiple recent reports [6, 7]. Further research in patients with a comparable clinical course, including correlation of thrombotic complications with IgG antibody titres post-vaccination, are essential in order to better understand the pathogenesis of such events.

The aim of our report is to provide a hypothesis of the possible course of events and underlying pathogenesis in this case of a young male with CRVO occurring soon after COVID-19 vaccination – the emphasis being on understanding various possibly interconnected phenomena, rather than conclusively establishing causality. We believe that the hypothesis we have proposed requires evidence in the form of prospective multi-centre studies to categorically prove association and causality.

## Data Availability

The datasets used and/or analyzed during the current study are available from the corresponding author on reasonable request.

## Abbreviations

CRVO: Central retinal vein occlusion (CRVO)
SARS-CoV-2: Severe acute respiratory syndrome coronavirus 2
VITT: Vaccine-induced immune thrombotic thrombocytopenia
OD: Right eye
OS: Left eye
BCVA: Best corrected visual acuity
OU: Both eyes
SD-OCT: Spectral-domain optical coherence tomography
CME: Cystoid macular edema
NSD: Neurosensory detachment
OCTA: Optical coherence tomography angiography
COVID-19: Coronavirus disease 2019
RT-PCR: Real-time reverse transcription–polymerase chain reaction
anti-PF4: Anti-platelet factor 4
anti-VEGF: Anti‑vascular endothelial growth factor
NF-κB: Nuclear factor kappa-light-chain-enhancer of activated B cells, Nuclear transcription factor-B
TLRs: Toll-like receptors
TTS: Thrombosis with thrombocytopenia syndrome
